# Prognostic signature associated with radioresistance in head and neck cancer via transcriptomic and bioinformatic analyses

**DOI:** 10.1186/s12885-018-5243-3

**Published:** 2019-01-14

**Authors:** Guo-Rung You, Ann-Joy Cheng, Li-Yu Lee, Yu-Chen Huang, Hsuan Liu, Yin-Ju Chen, Joseph T. Chang

**Affiliations:** 1 Graduate Institute of Biomedical Sciences, College of Medicine, Chang Gung University, Taoyuan, Taiwan; 2 Department of Medical Biotechnology and Laboratory Science, College of Medicine, Chang Gung University, Taoyuan, Taiwan; 3 Department of Radiation Oncology, Chang Gung Memorial Hospital-Linkou, Taoyuan, Taiwan; 4 Department of Pathology, Chang Gung Memorial Hospital-Linkou, Taoyuan, Taiwan; 5 Department of Oral Maxillofacial Surgery, Chang Gung Memorial Hospital-Linkou, Taoyuan, Taiwan; 6 Department of Biochemistry and Molecular Biology, College of Medicine, Chang Gung University, Taoyuan, Taiwan; 7 Molecular Medicine Research Center, Chang Gung University, Taoyuan, Taiwan; 8 Graduate Institute of Biomedical Materials and Tissue Engineering, College of Biomedical Engineering, Taipei Medical University, Taipei, Taiwan; 9 School of Biomedical Engineering, College of Biomedical Engineering, Taipei Medical University, Taipei, Taiwan; 10 Department of Radiation Oncology, Xiamen Chang Gung Memorial Hospital, Xiamen, Fujian China

**Keywords:** Head and neck cancer, Radioresistance, Prognosis, IGF1R, LAMC2, ITGB1, IL-6

## Abstract

**Background:**

Radiotherapy is an indispensable treatment modality in head and neck cancer (HNC), while radioresistance is the major cause of treatment failure. The aim of this study is to identify a prognostic molecular signature associated with radio-resistance in HNC for further clinical applications.

**Methods:**

Affymetrix cDNA microarrays were used to globally survey different transcriptomes between HNC cell lines and isogenic radioresistant sublines. The KEGG and Partek bioinformatic analytical methods were used to assess functional pathways associated with radioresistance. The SurvExpress web tool was applied to study the clinical association between gene expression profiles and patient survival using The Cancer Genome Atlas (TCGA)-head and neck squamous cell carcinoma (HNSCC) dataset (*n* = 283). The Kaplan-Meier survival analyses were further validated after retrieving clinical data from the TCGA-HNSCC dataset (*n* = 502) via the Genomic Data Commons (GDC)-Data-Portal of National Cancer Institute. A panel maker molecule was generated to assess the efficacy of prognostic prediction for radiotherapy in HNC patients.

**Results:**

In total, the expression of 255 molecules was found to be significantly altered in the radioresistant cell sublines, with 155 molecules up-regulated 100 down-regulated. Four core functional pathways were identified to enrich the up-regulated genes and were significantly associated with a worse prognosis in HNC patients, as the modulation of cellular focal adhesion, the PI3K-Akt signaling pathway, the HIF-1 signaling pathway, and the regulation of stem cell pluripotency. Total of 16 up-regulated genes in the 4 core pathways were defined, and 11 over-expressed molecules showed correlated with poor survival (TCGA-HNSCC dataset, *n* = 283). Among these, 4 molecules were independently validated as key molecules associated with poor survival in HNC patients receiving radiotherapy (TCGA-HNSCC dataset, *n* = 502), as IGF1R (*p* = 0.0454, HR = 1.43), LAMC2 (*p* = 0.0235, HR = 1.50), ITGB1 (*p* = 0.0336, HR = 1.46), and IL-6 (*p* = 0.0033, HR = 1.68). Furthermore, the combined use of these 4 markers product an excellent result to predict worse radiotherapeutic outcome in HNC (*p* < 0.0001, HR = 2.44).

**Conclusions:**

Four core functional pathways and 4 key molecular markers significantly contributed to radioresistance in HNC. These molecular signatures may be used as a predictive biomarker panel, which can be further applied in personalized radiotherapy or as radio-sensitizing targets to treat refractory HNC.

**Electronic supplementary material:**

The online version of this article (10.1186/s12885-018-5243-3) contains supplementary material, which is available to authorized users.

## Background

Head and neck cancer (HNC) is one of the most prevalent cancers worldwide [1–3]. The standard treatment for patients with HNC is surgery, radiation, chemotherapy or a combination of these treatments [4, 5]. Although treatment strategies have advanced in the last two decades, the overall 5-year survival rate for patients has not significantly changed. Tumor recurrence after radiotherapy is a major obstacle to recovery in HNC [4, 5]. The identification of radioresistant molecules contributing to a poor prognosis may facilitate patient consulting to determine proper treatment selection to improve the therapeutic outcome.

Previous studies have globally surveyed human genes associated with radioresistance in various cancers. Thus far, different experimental approaches have been used, including the direct comparison of two sets of samples with different levels of radiosensitivity in cancer tissues or cell lines [6–8]. A major disadvantage of this approach lies in the heterogeneity of the samples; the different radiosensitivities among various genetic backgrounds or tissue origins may also have a confounding effect on other pathological phenotypes. The use of microarrays to compare gene expression profiles between parental and radiation-treated cells has also been reported [9, 10]. Although this approach minimal the cause of genetic variation between individuals, those reports on the cellular and molecular effects often used short-term treatments of irradiation as a research model and may reflect the external radiation-induction situation. To recapitulate the condition of patients with intrinsic radioresistance, we have established several isogenic HNC cell sublines by long-term and low-dose serial irradiation [11, 12]. These subline cells have been shown possessing higher radioresistant phenotype compared to their parental cells [11, 12]. Similar approach has also been employed and demonstrated to efficiently generate high radioresistant cells [13–16].

To obtain a more comprehensive profile of the molecular network associated with radioresistance, in this study, we performed cDNA microarrays to globally survey differential transcriptomes between HNC cells and isogenic radioresistant sublines. We also used bioinformatic software to assess core molecular pathways associated with radioresistance. Through data mining of gene expression profiles available in the public domain, we further identified the biomarker signatures that contribute to the prognosis of HNC. Our study provides predictive or prognostic information, which may be further applied as biomarker criteria regarding treatment choices for individualized therapy in HNC.

## Methods

### Establishment of highly radioresistant HNC cell sublines

Three HNC cell lines, OECM1, FaDu and Detroit, were used [12, 13]. The cells were grown in MEM or RPMI 1640 medium supplemented with 10% fetal bovine serum, as previously described [12, 13]. The serial irradiation method was used to establish cell sublines with a highly radioresistant capability, similar to that described previously [12]. In principle, the long-term fractionated irradiation method was employed to select radioresistant subclone. Briefly, when cell confluence reached 50%, cells were treated with 2 Gy of radiation and continuously cultured. When they reached to approximate 85% confluence, the cells were trypsinized and subcultured into new plates. When they grew to 50% confluence, the cells were treated with another 2 Gy of radiation. The cells were continuously cultured and repeatedly treated until 60 Gy of irradiation was reached. This method has been previously demonstrated to successfully generate a more radioresistant sublines compared to the parental cells [11, 12]. In our HNC cells, it took approximately 3 months to complete the irradiation course. These subline cells (OECM1-RR, FaDu-RR, Detroit-RR) were harvested, examined for radiosensitivity, and subjected to cDNA microarray analysis. As shown, all these sublines exhibited higher radioresistance compared to their parent cells (Additional file 1: Figure S1).

### Profiling of radioresistant associated genes in HNC cells.

To globally determine the gene expression profile associated with radioresistance in HNC, cDNA microarray analysis was used to compare the different transcriptomes between 3 HNC parental cell lines and their radioresistant sublines. RNA extraction and cDNA microarray analysis were performed, similarly as previously described [17]. Briefly, total RNA was extracted by TRIzol reagent (Gibco BRL), and the quality and quantity were confirmed using an Agilent 2100 bioanalyzer (Agilent Technologies, Santa Clara, CA). The complementary RNA was hybridized to the Human Affymetrix U133A microarray gene chip and scanned by an Affymetrix GeneArray 2500 scanner (Affymetrix, Santa Clara, CA). Partek Genomics Suite software version 6.6 (Partek, St. Louis, MO) was used to normalize all signal intensities by RMA and to identify the gene expression levels of the microarray data. ANOVA was used to select the genes with a *p-*value < 0.05 and an average difference in expression of > 1.5-fold between parental and radioresistant cells. Hierarchical cluster analysis was applied to assess the similarity between sample groups.

### Analysis of radioresistant-associated molecular pathways in HNC

The molecular pathways of the differentially expressed genes identified in the microarray assays were analyzed using the Kyoto Encyclopedia of Genes and Genomes (KEGG) and Partek pathway (Partek, St. Louis, MO) analytical methods. KEGG is a collection of databases to provide molecular-level information to understand the functions and utilities of model biological systems [18]. KEGG Orthology groups (KO) analysis was used to identify molecular interaction networks. The computational platform of the Partek pathway algorithm (Partek Genomics Suite software 6.6) was used to identify the significant pathways for the differentially expressed genes according to the KEGG database. The pathways with a *p*-value < 0.05 were selected as statistically significant candidates.

### Identification of radioresistance-associated genes that contribute to a poor prognosis in HNC patients

The prognostic effect of radioresistance-associated genes in HNC patients was evaluated using the SurvExpress web tool [19]. SurvExpress is a versatile online biomarker validation tool to assess the significance of multi-gene expression in various cancers using survival analysis. The dataset of The Cancer Genome Atlas (TCGA) head and neck cancer cohort (*n* = 283) was used in this study. For SurvExpress analysis, high- and low-risk groups were classified by the optimization algorithm from the order of the prognostic index according to each gene expression level. The high-risk group was selected to perform Kaplan-Meier analysis for overall survival, and the log-rank test was used to calculate hazard ratios (HRs) and their 95% confidence intervals (CIs).

To further validate the prognostic value of the genes in HNC patients receiving radiotherapy, a larger-scale, head and neck cancer cohort (*n* = 502) was used. The gene expression levels of the TCGA-HNSCC cohort (level 3, RNASeqv2 RSEM genes normalized data) were obtained from Broad GDAC firehose (gdac.broadinstitute.org). Clinical information about this cohort was retrieved from the Genomic Data Commons (GDC)-Data-Portal of National Cancer Institute (portal.gdc.cancer.gov). In this cohort, 335 HNC patients who received radiotherapy were recruited for prognostic analyses by using log-rank test and Kaplan-Meier survival method.

To extend the potential application of the candidate molecules, the prognostic significance of the combined markers was further evaluated. The cutoff point of high or low- expression was defined as the medium expression value of each candidate molecule. The high-risk group was defined as the patients who possessed at least one markers with high-expressions, while low-risk group being the patients without any high-level marker. The log-rank test and Kaplan-Meier survival method were used to examine prognostic significance. All *P* values were two-sided, and the significance level was set at *P* < 0.05.

## Results

### Gene expression profile associated with radioresistance in HNC cells

To obtain a comprehensive profile of molecules that may represent the intrinsic factor of radioresistance in HNC, we established 3 radioresistant (RR) sublines derived from HNC cancer cell lines. The cDNA microarray database was established by comparing the mRNA expression profiles between the parental HNC cells and RR subline cells. After ANOVA by the criteria of a *p*-value < 0.05 and average expression change > 1.5-fold between parental and RR sublines, 255 genes (294 probe-sets) were filtered out. Unsupervised hierarchical clustering analysis of these 255 genes classified the cells into two groups (parental and RR), in which 155 genes were up-regulated and 100 were down-regulated in the RR sublines (Additional file 1: Figure S2). Additional file 2: Table S1 and Table S2 list the top 100 up-regulated and top 50 down-regulated genes differentially expressed in the RR cells. These genes represent the expression profile associated with radioresistance in HNC cells.

### Core molecular pathways associated with radioresistance in HNC cells

**Fig. 1:**
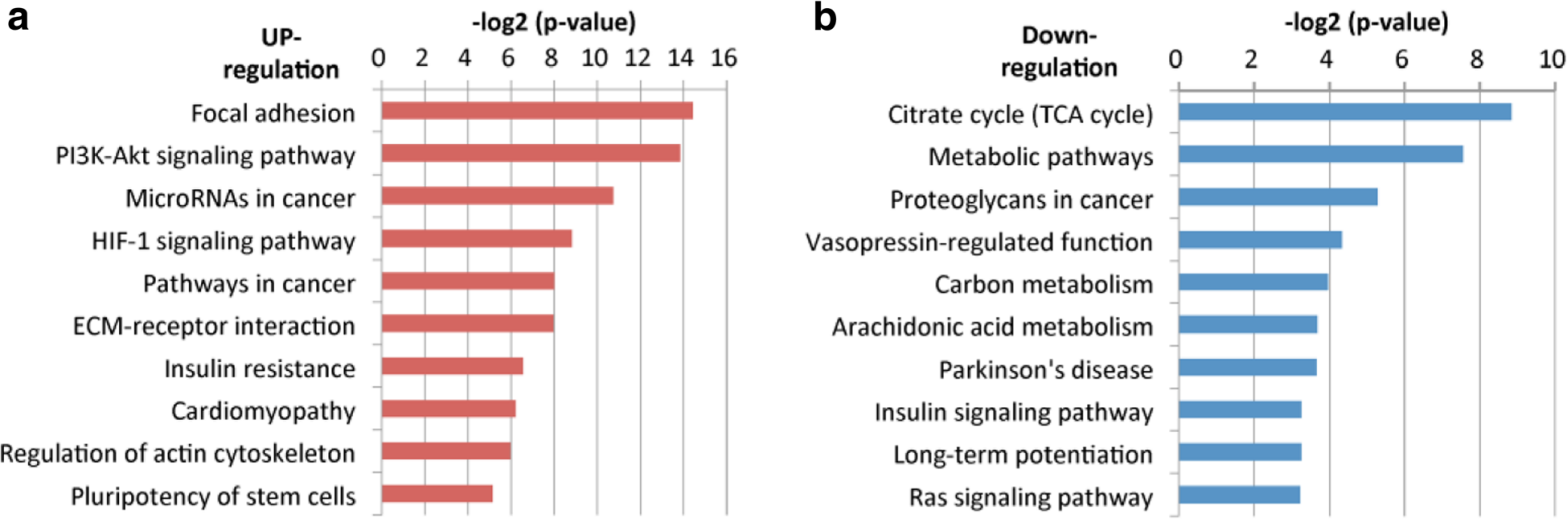
Bioinformatics analysis of the functional pathway contributing to radioresistance in HNC cells. **(a**) A list of the top 10 significant molecular pathways determined by KEGG pathway enrichment analysis for 155 up-regulatory genes. (**b**) A list of the top 10 significant molecular pathways determined by KEGG pathway enrichment analysis for the 100 down-regulatory genes

To obtain a global picture of the molecular pathways that may contribute to radioresistance, the 155 up-regulated and 100 down-regulated genes were imported into the KEGG KO suite for integrated network analysis. With the association of the up-regulatory mechanism, these 155 molecules were found to be enriched in the pathways related to oncogenic functions in general. Among the top 10 significant pathways, cell mobility comprised 3, as the regulation of focal adhesion, extracellular matrix (ECM), and cytoskeleton. Other oncogenic mechanisms, including the PI3K-Akt signaling pathway, HIF-1 signaling pathway, and pluripotency of stem cells, were also noted (Fig. 1a). Regarding the association of a down-regulatory mechanism, 100 molecules were found to be enriched in the pathways mostly associated with cellular metabolism. These include the TCA cycle, proteoglycans in cancer, carbon metabolism, and arachidonic acid metabolism (Fig. 1b). To identify the molecular signature contributing to radioresistance in HNC, the oncogenic associated pathways were addressed, as the modulation of focal adhesion (*p* = 0.00005), PI3K-Akt signaling (*p* = 0.00007), HIF-1 signaling (*p* = 0.00223), and pluripotency of stemness (*p* = 0.02831).

### Prognostic significance of the 4 core pathways in HNC patients

The prominent molecules associated with radioresistance were focus on the 4 core oncogenic pathways. Among the 155 up-regulated genes, 11, 14, 5, and 5 molecules were enriched in the focal adhesion, PI3K-Akt, HIF-1, and stemness pathways, respectively (Table 1). Aside from the overlapped molecules, 16 RR-associated genes existed in these core pathways, indicating the high significance of these genes to facilitate radioresistance in HNC.

**Table 1:** List of up-regulated genes enriched in the four core molecular pathways

KEGG pathway name	Gene list
Focal adhesion	FLNA, FLNB^a^, GSK3B, IGF1R^a^, ITGA6^a^, ITGB1^a^, ITGB4^a^, LAMA3^a^, LAMC2^a^, MYL9^a^, VEGFA^a^
PI3K-Akt signaling pathway	DDIT4^a^, GNG4, EFNA1, FGFR3, GSK3B, IGF1R^a^, ITGA6^a^, ITGB1^a^, ITGB4^a^, IL6^a^, LAMA3^a^, LAMC2^a^, SGK1^a^, VEGFA^a^
HIF-1 signaling pathway	ENO2^a^, IGF1R^a^, IL6^a^, SLC2A1^a^, VEGFA^a^
Pluripotency of stem cells	FGFR3, FZD10^a^, GSK3B, IGF1R^a^, JARID2^a^

^a^Top 100 up-regulated genes

**Fig. 2:**
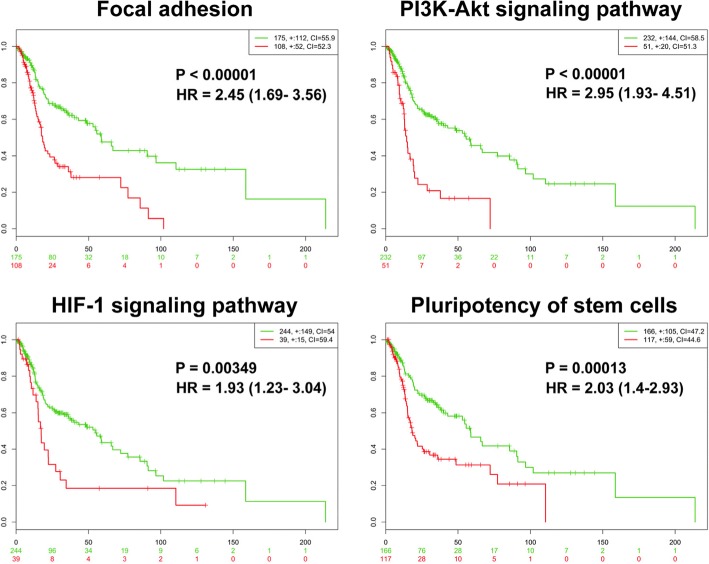
Prognostic significance of the 4 core functional pathways (focal adhesion, PI3K-Akt signaling, HIF-1 signaling, and pluripotency of stem cells) in HNC patients, as determined by SurvExpress analysis from the TCGA-HNSCC dataset (*n* = 283). For each pathway, the log-rank test of the Kaplan-Meier survival curve for the risk group, the hazard ratio (HR) and the *p*-value (P) are shown

To determine the clinical significance of these core pathways, we investigated the association between the gene expression levels of these 16 molecules in HNC patients (grouping into 4 specific pathways) and the patient survival status. The TCGA cohort (HNC, *n* = 283) was used via the SurvExpress analytical method. The results are shown in Fig. 2. As shown, all the core pathways exhibited a prominent correlation with poor survival, as the focal adhesion (*p* < 0.00001, HR = 2.45), PI3K-Akt signaling (*p* < 0.00001, HR = 2.95), HIF-1 signaling (*p* < 0.00349, HR = 1.93), and pluripotency of stem cells (*p* = 0.00013, HR = 2.03). These results suggested that these RR-associated core pathways could distinguish among patients in the high-risk group and predict a poor prognosis in HNC.

### Prognostic significance of the panel of radioresistant genes in HNC patients

**Fig. 3:**
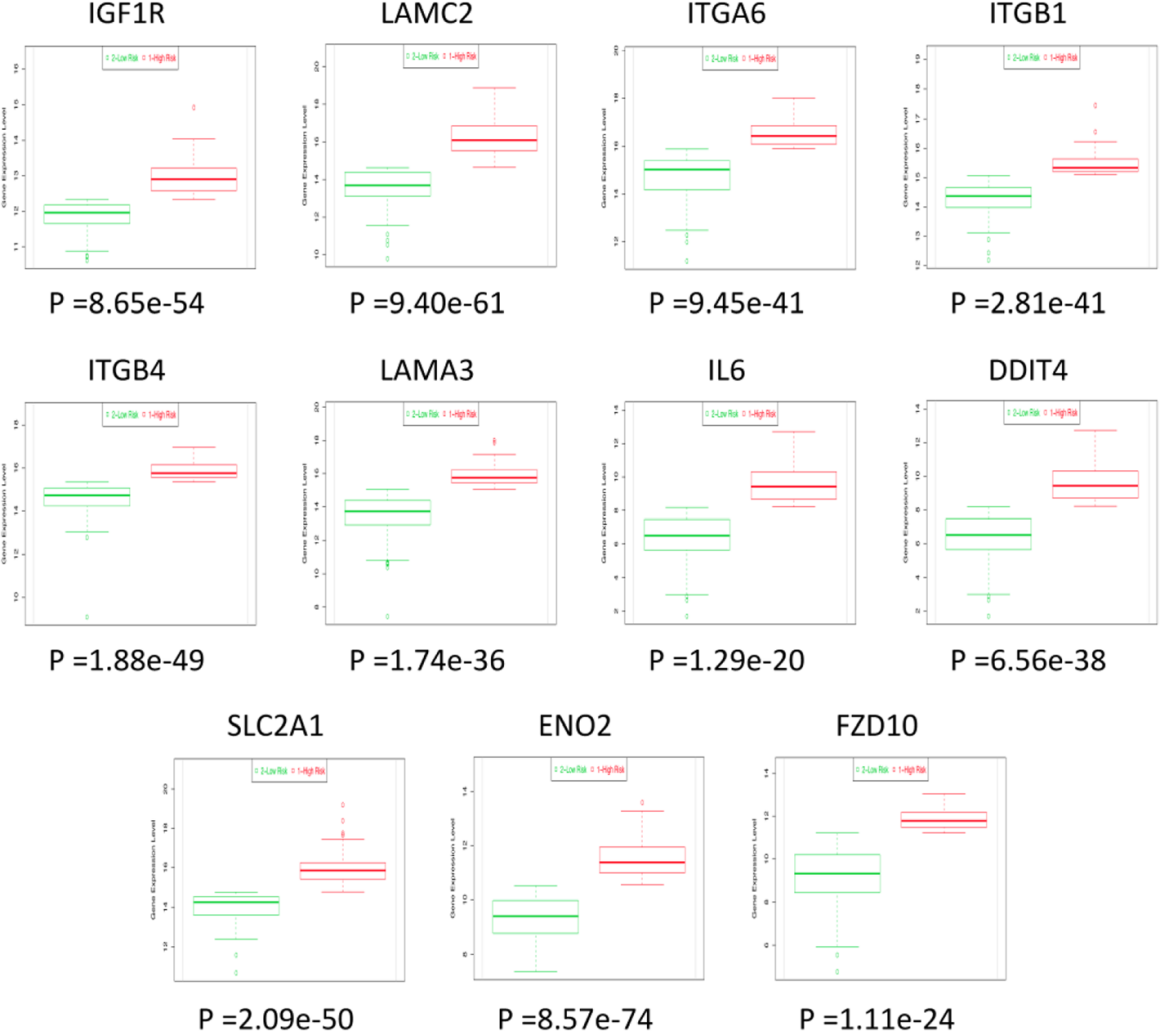
Differential expression of 11 selected genes between low- and high-risk groups of HNC patients, as determined by SurvExpress analysis from the TCGA-HNSCC dataset (n = 283)

To assess the clinical significance of the 16 RR-associated genes in HNC, we investigated the association between each gene expression and patient survival status using the TCGA cohort (HNC, *n* = 283) and SurvExpress analytical method. For each gene, the *p*-value and HR are summarized in Table 2. As shown, 11 of the genes exhibited over-expression in the cancer tissues and were statistically correlated with poor survival (*p* < 0.05): IGF1R, LAMC2, ITGA6, ITGB1, ITGB4, LAMA3, IL-6, DDIT4, SLC2A1, ENO2, and FZD10 (Fig. 3). Thus, this panel of molecules represents molecular biomarkers to predict a poor prognosis in HNC patients.

**Table 2:** Prognostic significance of the 16 selected genes in the HNC patients

No.	Gene	*P* -value	Hazard ratio (CI)	Pathway
4	IGF1R	0.03497	1.57 (1.03–2.41)	Focal adhesion HIF-1 signaling pathway PI3K-Akt signaling pathway Pluripotency of stem cells
3	VEGFA	0.14780	1.31 (0.91–1.88)	Focal adhesion HIF-1 signaling pathway PI3K-Akt signaling pathway
2	LAMC2	0.00228	1.97 (1.26–3.07)	Focal adhesion PI3K-Akt signaling pathway
2	ITGA6	0.00514	1.76 (1.10–2.64)	Focal adhesion PI3K-Akt signaling pathway
2	ITGB1	0.01010	1.80 (1.14–2.83)	Focal adhesion PI3K-Akt signaling pathway
2	ITGB4	0.01463	1.57 (1.09–2.27)	Focal adhesion PI3K-Akt signaling pathway
2	LAMA3	0.01549	1.69 (1.10–2.60)	Focal adhesion PI3K-Akt signaling pathway
2	IL6	0.01611	1.57 (1.08–2.28)	HIF-1 signaling pathway PI3K-Akt signaling pathway
1	DDIT4	0.00002	2.45 (1.61–3.72)	PI3K-Akt signaling pathway
1	JARID2	0.00212	1.91 (1.26–2.91)	Pluripotency of stem cells
1	SLC2A1	0.00421	2.17 (1.26–3.73)	HIF-1 signaling pathway
1	ENO2	0.00447	1.69 (1.17–2.44)	HIF-1 signaling pathway
1	FZD10	0.03798	1.71 (1.02–2.87)	Pluripotency of stem cells
1	MYL9	0.07662	0.65 (0.41–1.05)	Focal adhesion
1	FLNB	0.12240	1.33 (0.92–1.93)	Focal adhesion
1	SGK1	0.03847	1.64 (1.02–2.63)	PI3K-Akt signaling pathway

*CI* confidence intervals

### Prognostic signature for the prediction of a worse radiotherapeutic outcome in HNC

To further confirm the prognostic value of the RR genes in the patients who received radiotherapy, an independent validation study was performed. Of the 11 genes that exhibited significant values in the prediction of a poor prognosis, 7 involved in more than 2 core pathways (Table 2) were selected. To fast examine whether these 7 molecules screened from the smaller TCGA-HNSCC dataset (*n* = 283) may also exhibit significance in a larger TCGA-HNSCC dataset (*n* = 502), the combinational analysis of these markers with the survival status of the HNC patients were examined via SurvExpress analytical method. As shown, remarkable associations of these combine molecules in either TCGA-HNSCC datasets were found (Additional file 1: Figure S4). Thus, although different approaches may produce various results, the most prominent molecules usually still come out.

**Fig. 4:**
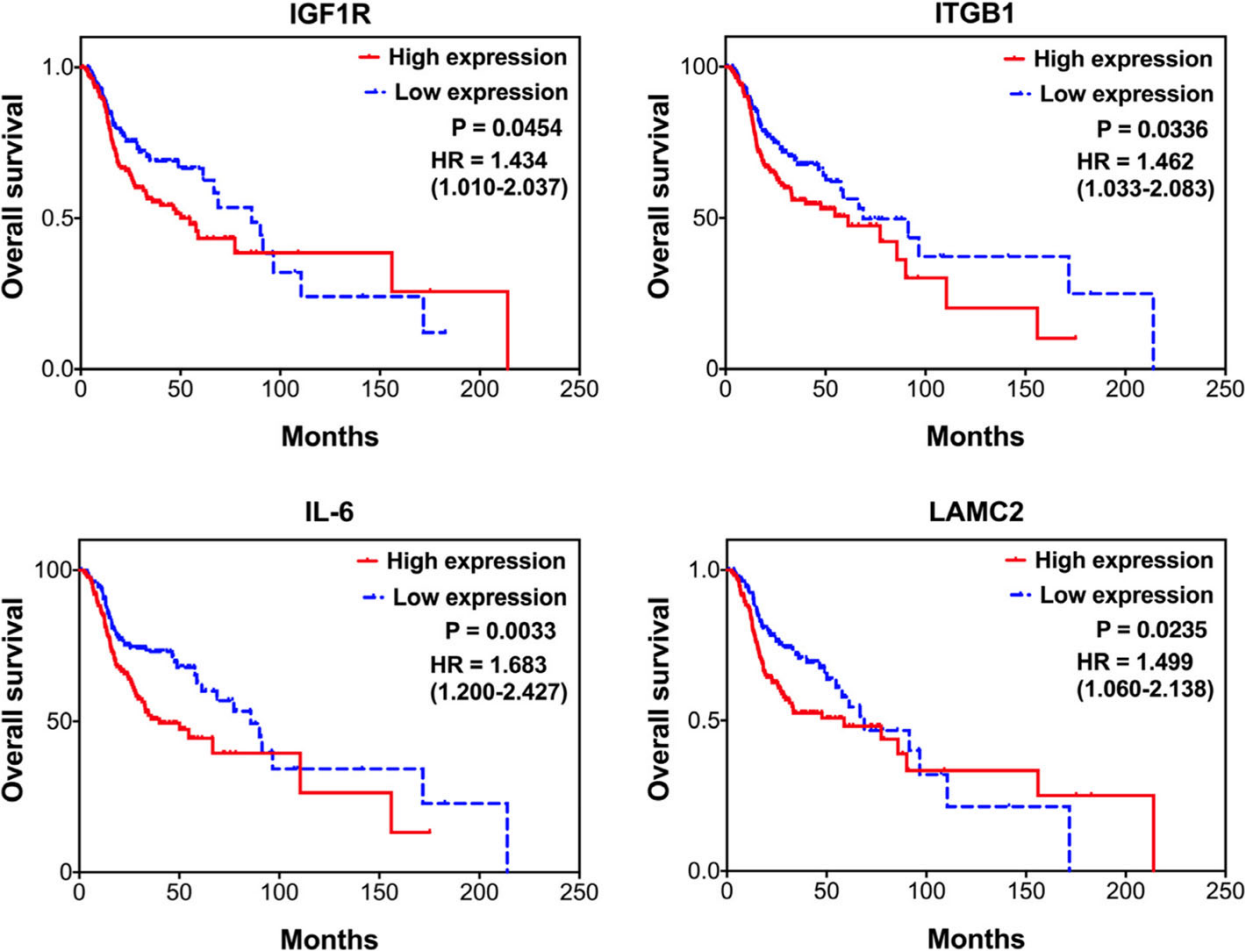
Prognostic significance of the 4 key molecules (IGF1R, LAMC2, ITGB1 and IL-6) in HNC patients receiving radiotherapy, as determined by Kaplan-Meier survival analysis from the TCGA-HNSCC dataset (*n* = 502). The clinical data were retrieved via the Genomic Data Commons (GDC)-Data-Portal of National Cancer Institute. For each gene, the survival curve, hazard ratio (HR) and *p*-value (P) are shown

In the larger TCGA-HNSCC dataset (*n* = 502), 335 patients who received radiotherapy were filtered out for validation study via Kaplan-Meier survival analytical method. As shown in Fig. 4, four molecules were significantly correlated with a poor overall survival, as IGF1R (*p* = 0.0454, HR = 1.43), LAMC2 (*p* = 0.0235, HR = 1.50), ITGB1 (*p* = 0.0336, HR = 1.46), and IL-6 (*p* = 0.0033, HR = 1.68). Consistently, these four molecules were confirmed up-regulated in the cellular RR sublines compared to their parental HNC cell lines (Attached file 1: Figure S3). Although ITGA6, ITGB4, and LAMA3 showed no statistical association with patient survival (*p* > 0.05), these molecules were found to be highly correlated with other significant molecules (Additional file 2: Table S3). Thus, these molecules may also play important roles in facilitating radioresistance in HNC.

**Fig. 5:**
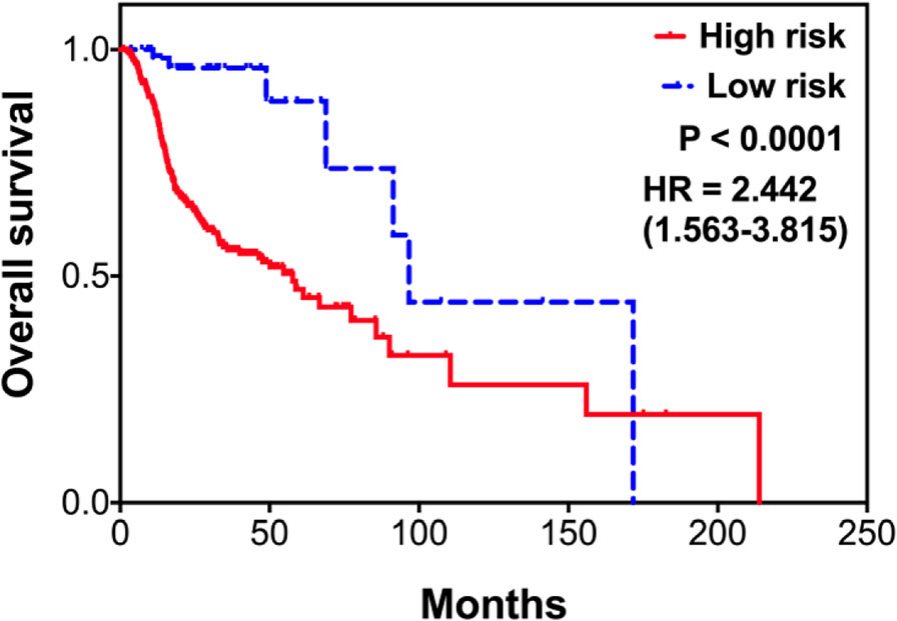
Prognostic effectiveness of the combined 4 markers in HNC patients receiving radiotherapy, as determined by Kaplan-Meier survival analysis from the TCGA-HNSCC dataset (*n* = 502). The patients possessed at least one high-level markers were defined as the high-risk group, while those without any high-level marker being low-risk group. The survival curve, hazard ratio (HR) and *p*-value (P) are shown

To enrich potential application of these 4 molecules, the effectiveness of prognostic prediction by the use of combined markers was further determined. For those 335 HNC patients receiving radiotherapy, 277 patients possessing at least one high-level marker was defined as high-risk group, while 58 without any high-level marker was defined as low-risk group. The Kaplan-Meier survival analysis was used to assess the prognostic significance. As shown in Fig. 5, this combined panel showed an excellent association with poor survival (*p* < 0.0001, HR = 2.44). This result suggested that the use of combined molecules gained an advantage of the individual marker to produce an outstanding prognostic efficacy. In all, we have identified 4 prognostic biomarkers, IGF1R, LAMC2, ITGB1, and IL-6, and demonstrated a combine panel of molecular signature to predict a worse radiotherapeutic outcome in HNC.

## Discussion

Radiotherapy is an indispensable treatment modality in HNC, while radioresistance is the major cause of treatment failure. Therefore, the identification of resistant molecules may allow further clinical applications in personalized radiotherapy. For this goal, we have obtained several important findings in this study. (1) Without interference by genetic heterogeneity, 255 genes were identified to be associated with radioresistance in HNC cells, including 155 up-regulated and 100 down-regulated genes. (2) Four core molecular pathways significantly contributed to radioresistance in HNC cell lines and HNC patients: modulation of cellular focal adhesion, the PI3K-Akt signaling pathway, HIF-1 signaling pathway, and pluripotency of stem cells. (3) Eleven molecules were associated with a poor survival in HNC, 4 of which, IGF1R, LAMC2, ITGB1, and IL-6, were key molecules to predict a worse prognosis of radiotherapy. Further validation studies are highly recommended to confirm these results in a subgroup of HNC patients, such as for a specific anatomic subsite, a specific ethic patient group, or for the cancers caused by a specific etiology.

Four core pathways played substantial roles related to radioresistance in HNC. In the pathway modulating focal adhesion, 11 RR genes were enriched (Tables 1 and 2). Molecules in the laminin (LAMA3, LAMC2) and integrin (ITGA6, ITGB1, ITGB4) families composed the majority in this pathway, indicating the close link between these signaling pathways and radioresistance. Laminin, a family of extracellular matrix glycoproteins, is the major non-collagenous constituent of the basement membrane. It comprises 3 chains, alpha, beta and gamma, to form various heterotrimeric laminin isoforms [20]. Integrin is a family of transmembrane receptors that facilitates extracellular matrix adhesion. It consists of alpha and beta subunits with several isoforms to form a heterodimeric protein [21]. Although the major functions of laminin and integrin are to maintain the mechanical integrity of the cell membrane and regulate cell mobility, recent studies have shown much more biological roles than what was originally thought. Through the interaction of laminin and its cell-surface receptors, including integrin, this complex protein activates signal transduction pathways that may mediate various cellular functions, including cell proliferation, differentiation, invasion, tumor angiogenesis and metastasis [21–23]. In this study, we further noted that up-regulation of this focal adhesion pathway plays an important role in facilitating radioresistance in HNC (Figs. 1 and 2). Our finding is consistent with recent reports that cell adhesion molecules, such as laminin and integrin family proteins, participate in radioresistance, chemoresistance, or cell survival in several cancers [24–28]. We also demonstrated that LAMC2, a laminin component, and ITGB1, an integrin subunit, exhibit excellent correlation with poor outcome in HNC patients receiving radiotherapy (Figs. 3 and 4). These results are supported by other investigations as well. LAMC2 is over-expressed in several types of cancers, including urothelial, lung, colorectal and head-neck cancers, and is associated with a poor prognosis [29–33]. Similarly, a high level of ITGB1 predicts poor survival in breast and gastric cancers [34–38]. Furthermore, LAMC2 or ITGB1 may serve as therapeutic targets via modulating molecular expression to improve radiosensitivity [39–42]. All these results highlight the importance of the laminin-integrin axis pathway in radioresistance and cancer aggressiveness.

Another pathway identified to be associated with radioresistance, the PI3K-Akt signaling pathway, was shown to be highly significant (Figs. 1 and 2). PI3K-Akt is an intracellular signaling pathway that promotes cell growth and survival in response to extracellular stimuli [43]. It is an important downstream mediator of several membrane-bound receptor tyrosine kinases. Hyper-activation of this pathway correlates with many aggressive cancer phenotypes, such as cell proliferation, tumor angiogenesis, metastasis, and a poor prognosis [43]. Furthermore, the PI3K-Akt pathway contributes to radioresistance in many types of cancers, a finding that was consistent with ours [44, 45]. In this study, we also noted that IL-6 is a key extracellular signaling molecule participating in the PI3K-Akt signaling pathway (Tables 1 and 2). IL-6 is a pleiotropic cytokine that is involved in multiple biological responses, including auto-immunity, inflammation and cancers [46, 47]. It can mediate numerous downstream effectors by activating several signaling cascades, including the JAK/STAT, MAPK, and PI3K/AKT pathways, to promote cancer progression [47–50]. Specifically, the function of IL-6 in therapeutic resistance has been commonly reported. This molecule can protect cells from radiation- or drug-induced DNA damage by suppressing oxidative stress or the induction of anti-apoptotic mechanism to facilitate cell survival [50–53]. Clinically, IL-6 has also been found to be over-expressed in almost all types of tumors and is associated with a poor prognosis of cancers such as cervical, lung, gallbladder, and head-neck cancers [54–58]. All these reports support our findings that IL-6 is a critical factor that is over-expressed in cancers and regulates the PI3K-Akt signaling pathway, leading to radioresistance and a worse treatment outcome.

The pathways of cancer stemness and HIF-1 signaling exhibited a prominent function in this study (Figs. 1 and 2). Recently, accumulating studies have shown that cancer stem cells, a subset of cancer cells, possess stem cell-like properties, which may serve as the driving force for tumorigenesis. These cells have a strong malignant potential, with self-renewal capacity, high mobility, and stress tolerance, resulting in resistance to chemo-radiotherapy [59, 60]. The HIF-1 signaling pathway is critical in the maintenance of the cancer stemness phenotype, which may be induced by the hypoxia condition in the tumor microenvironment [61–63]. This signaling pathway is also an important factor contributing to radioresistance in many cancers [64, 65]. All these reports support our findings, suggesting that the intra-tumor hypoxia condition induces the HIF-1 signaling pathway, further facilitating cancer stemness formation and resulting in radioresistance in HNC. We also noted that IGF1R is an important molecule participating in the HIF-1 signaling pathway and regulating cancer stemness (Tables 1 and 2). These results are consistent with previous studies by other investigators [66–69]. IGF1R has tyrosine kinase activity, which plays a significant role during cancer cell transformation mainly via cytoprotection and anti-apoptosis [70–72]. Apparently, high expression of this molecule in tumors confers resistance to chemo-radiotherapy [70–72]. Consistently, we found that IGF1R is over-expressed in cancer tissues and is associated with adverse outcomes in HNC patients receiving radiotherapy (Figs. 3 and 4). Similar results were also found in many types of cancers, including prostate, lung, ovary and renal cancers [73–77]. Taken together, our results demonstrated that IGF1R is an unfavorable prognostic marker in HNC patients treated with radiotherapy. This may result from modulation of the HIF-1 signaling pathway, leading to stem cell conversion and a radioresistant phenotype.

## Conclusions

Multi-gene biomarker is one of the most important issues for clinical application. In this study, we have identified 255 genes significantly associated with radioresistance in HNC through global survey techniques. Via data mining and bioinformatic analyses, we have addressed 4 signaling pathways and 11 molecules associated with poor survival in HNC. Among these molecules, 4 markers LAMC2, ITGB1, IL-6, and IGF1R were demonstrated to form a panel marker to predict a worse radiotherapeutic outcome. This panel signature can be applied as predictive information for radiotherapy and may be further used to develop radio-sensitizing modalities for the treatment of refractory HNC.

## Additional files

Additional file 1: **Figure S1.** Verification of the radioresistant phenotype of radioresistant sublines. A total of 500 cells parental (OECM1-Pt, Detroit-Pt, FaDu-Pt) or radioresistant subline cells (OECM1-RR, Detroit-RR, and FaDu-RR) were seeded per well in a 96-well plate and following treated irradiation with various doses (0, 3, and 6 Gy) and continuously cultured for 4 days. The cell survival fractions were assessed using Cell Counting Kit-8. Results were presented as the mean ± standard deviation (SD) from three independent experiments. ***, *P* < 0.0001. **Figure S2.** Hierarchical clustering analysis of the gene expression profiles among the three HNC cell lines (OECM1, FaDu, Detroit) and their radioresistant (RR) sublines. **Figure S3.** Differential expressions of 4 marker proteins (IFG1R, LAMC2, ITGB1, and IL6) between HNC cell lines and their radioresistant sublines. Three HNC parental cells (OECM1-Pt, Detroit-Pt, FaDu-Pt) and their radioresistant subline cells (OECM1-RR, Detroit-RR, and FaDu-RR) were used. The IGF1R, LAMC2, TGB1, and GAPDH were determined from total cell lysate, whereas IL-6 was determined from serum- free conditioned medium (CM). The relative expression of protein was normalized to GAPDH in each individual sample. Over-expression of these four proteins were found in the radioresistant subline cells as revealed by Western blotting analysis. **Figure S4.** Prognostic significance of the combined 7 RR molecules (IGF1R, LAMC2, ITGA6, ITGB1, ITGB4, LAMA3 and IL6) in HNC patients, as determined by SurvExpress analysis from two different TCGA-HNSCC datasets (*n* = 283, and *n* = 502). In each dataset, the log-rank test of the Kaplan-Meier survival curve, the hazard ratio (HR) and the *p*-value (P) are shown. (DOCX 1398 kb)

Additional file 2: **Table S1.** The top 100 up-regulated genes associated with radioresistance in HNC cells. **Table S2.** The top 50 down-regulated genes associated with radioresistance in HNC cells. **Table S3.** Correlative expressions of the 7 molecules in HNC patients receiving radiotherapy. (DOCX 42 kb)

## Abbreviations

CIs: Confidence intervals
HNC: Head and neck cancer
HNSCC: Head and neck squamous cell carcinoma
HR: Hazard ratio
KEGG: Kyoto encyclopedia of genes and genomes
RR: Radioresistant
TCGA: The cancer genome atlas
