# Supplementary material for: Prognostic signature associated with radioresistance in head and neck cancer via transcriptomic and bioinformatic analyses
Source: BMC Cancer. 2019 Jan 14;19:64. doi: 10.1186/s12885-018-5243-3 (PMC6332600; doi:10.1186/s12885-018-5243-3)
Supplement: Supplementary file 1 — Figure S1. Verification of the radioresistant phenotype of radioresistant sublines. A total of 500 cells parental (OECM1-Pt, Detroit-Pt, FaDu-Pt) or radioresistant subline cells (OECM1-RR, Detroit-RR, and FaDu-RR) were seeded per well in a 96-well plate and following treated irradiation with various doses (0, 3, and 6 Gy) and continuously cultured for 4 days. The cell survival fractions were assessed using Cell Counting Kit-8. Results were presented as the mean ± standard deviation (SD) from three independent experiments. ***, P < 0.0001. Figure S2. Hierarchical clustering analysis of the gene expression profiles among the three HNC cell lines (OECM1, FaDu, Detroit) and their radioresistant (RR) sublines. Figure S3. Differential expressions of 4 marker proteins (IFG1R, LAMC2, ITGB1, and IL6) between HNC cell lines and their radioresistant sublines. Three HNC parental cells (OECM1-Pt, Detroit-Pt, FaDu-Pt) and their radioresistant subline cells (OECM1-RR, Detroit-RR, and FaDu-RR) were used. The IGF1R, LAMC2, TGB1, and GAPDH were determined from total cell lysate, whereas IL-6 was determined from serum- free conditioned medium (CM). The relative expression of protein was normalized to GAPDH in each individual sample. Over-expression of these four proteins were found in the radioresistant subline cells as revealed by Western blotting analysis. Figure S4. Prognostic significance of the combined 7 RR molecules (IGF1R, LAMC2, ITGA6, ITGB1, ITGB4, LAMA3 and IL6) in HNC patients, as determined by SurvExpress analysis from two different TCGA-HNSCC datasets (n = 283, and n = 502). In each dataset, the log-rank test of the Kaplan-Meier survival curve, the hazard ratio (HR) and the p-value (P) are shown. (DOCX 1398 kb) [file 12885_2018_5243_MOESM1_ESM.docx]

**Additional file 1.**

**Fig S1.** Verification of the radioresistant phenotype of radioresistant sublines. A total of 500 cells parental (OECM1-Pt, Detroit-Pt, FaDu-Pt) or radioresistant subline cells (OECM1-RR, Detroit-RR, and FaDu-RR) were seeded per well in a 96-well plate and following treated irradiation with various doses (0, 3, and 6 Gy) and continuously cultured for 4 days. The cell survival fractions were assessed using Cell Counting Kit-8. Results were presented as the mean±standard deviation (SD) from three independent experiments. ***, P<0.0001.

**
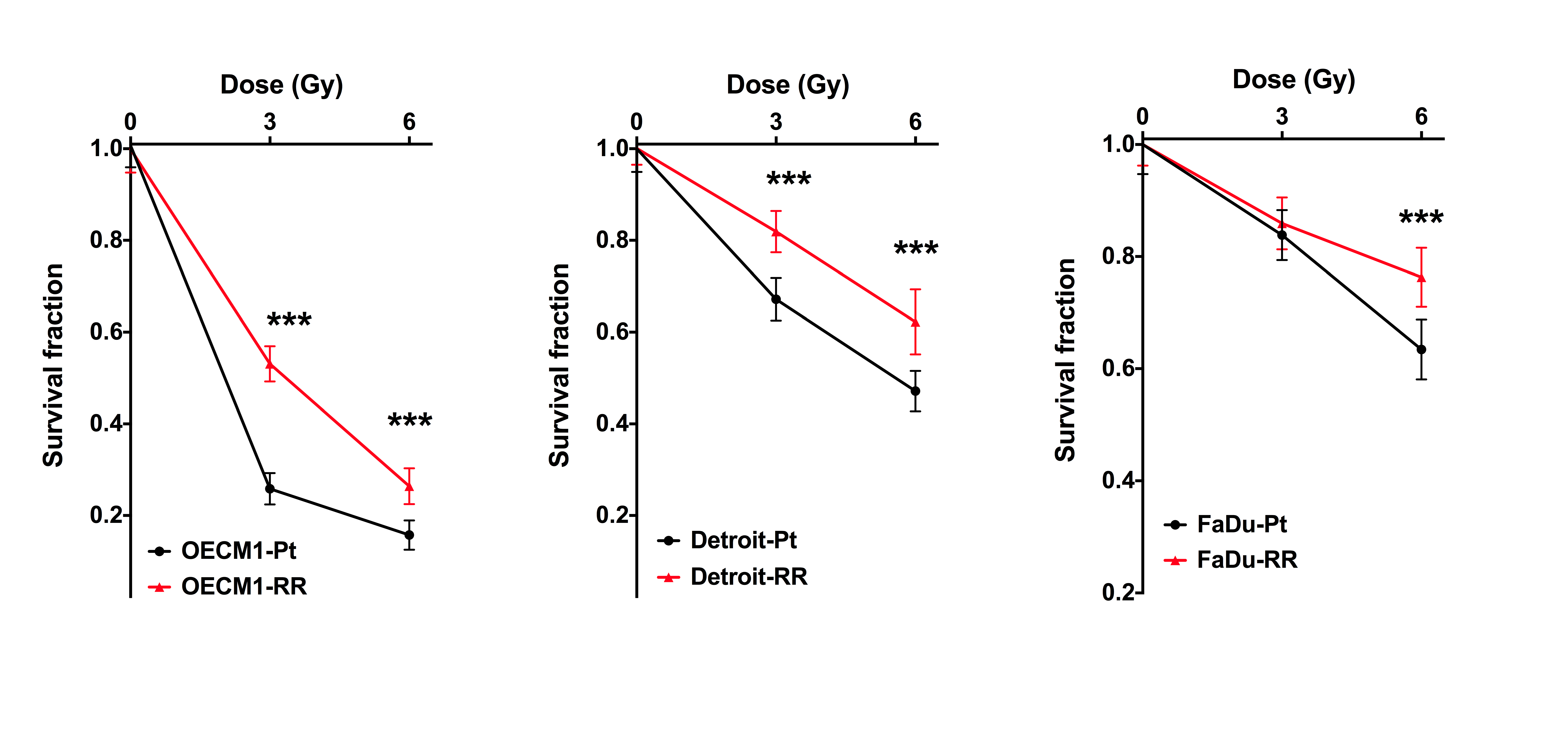
**

**Fig S2.** Hierarchical clustering analysis of the gene expression profiles among the three HNC cell lines (OECM1, FaDu, Detroit) and their radioresistant (RR) sublines.

**
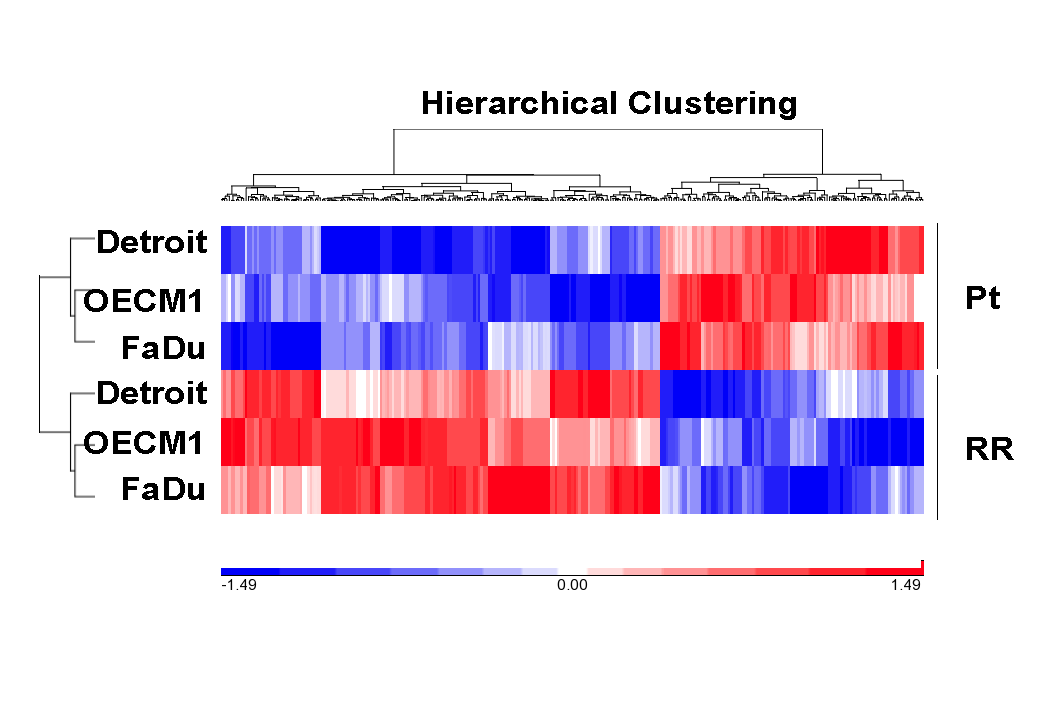
**

**Fig S3.** Differential expressions of 4 marker proteins (IFG1R, LAMC2, ITGB1, and IL6) between HNC cell lines and their radioresistant sublines. Three HNC parental cells (OECM1-Pt, Detroit-Pt, FaDu-Pt) and their radioresistant subline cells (OECM1-RR, Detroit-RR, and FaDu-RR) were used. The IGF1R, LAMC2, TGB1, and GAPDH were determined from total cell lysate, whereas IL-6 was determined from serum- free conditioned medium (CM). The relative expression of protein was normalized to GAPDH in each individual sample. Over-expression of these four proteins were found in the radioresistant subline cells as revealed by Western blotting analysis.

**
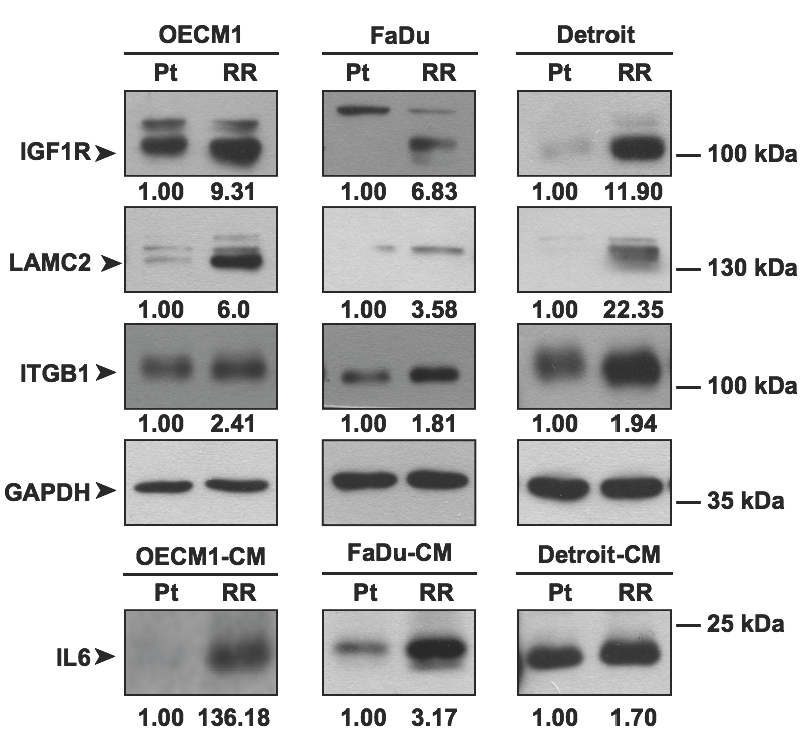
**

**Fig S4.** Prognostic significance of the combined 7 RR molecules (IGF1R, LAMC2, ITGA6, ITGB1, ITGB4, LAMA3 and IL6) in HNC patients, as determined by SurvExpress analysis from two different TCGA-HNSCC datasets (n=283, and n=502). In each dataset, the log-rank test of the Kaplan-Meier survival curve, the hazard ratio (HR) and the *p*-value (P) are shown.

**
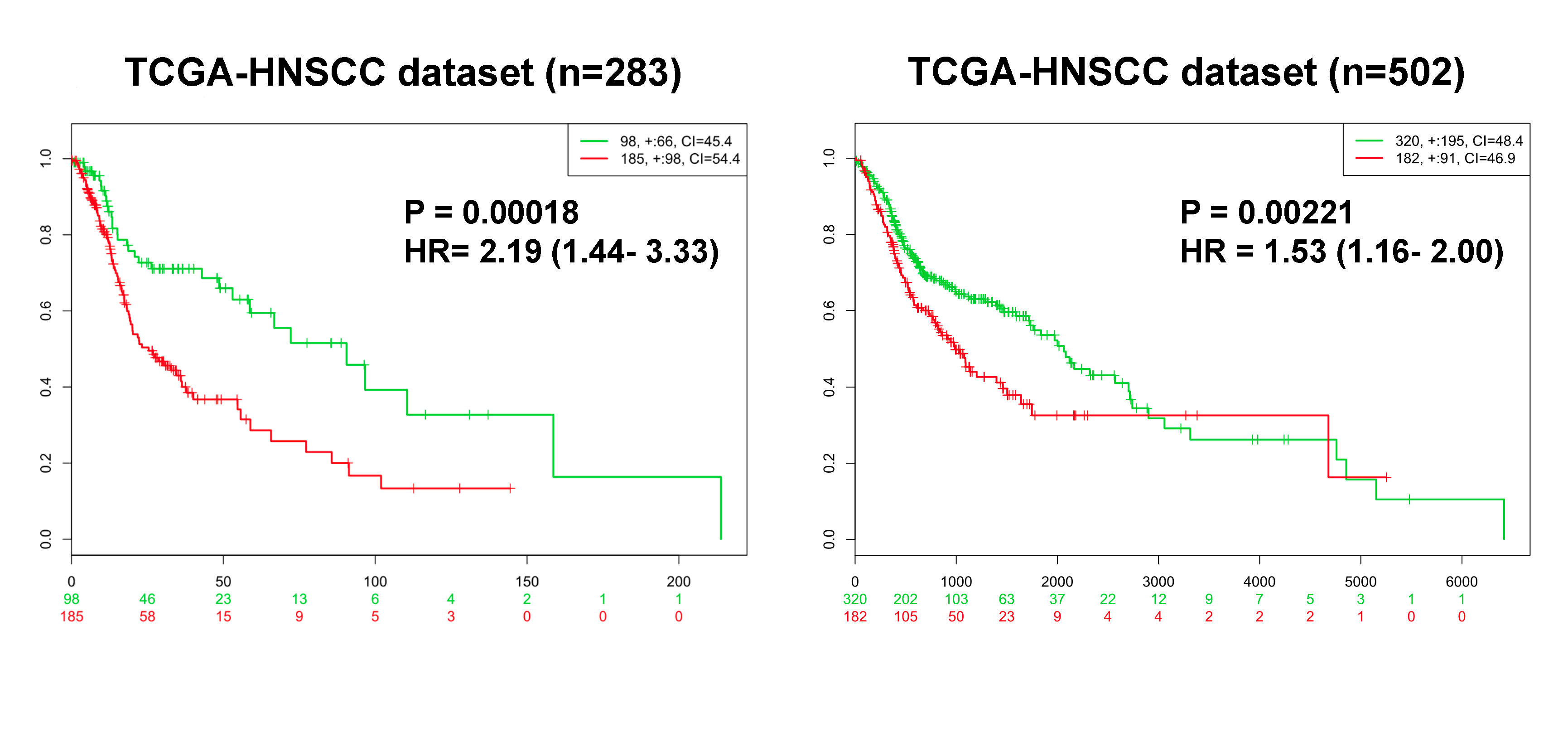
**
