# Supplementary material for: Prognostic signature associated with radioresistance in head and neck cancer via transcriptomic and bioinformatic analyses
Source: BMC Cancer. 2019 Jan 14;19:64. doi: 10.1186/s12885-018-5243-3 (PMC6332600; doi:10.1186/s12885-018-5243-3)
Supplement: Supplementary file 2 — Table S1. The top 100 up-regulated genes associated with radioresistance in HNC cells. Table S2. The top 50 down-regulated genes associated with radioresistance in HNC cells. Table S3. Correlative expressions of the 7 molecules in HNC patients receiving radiotherapy. (DOCX 42 kb) [file 12885_2018_5243_MOESM2_ESM.docx]

**Additional file 2**

**Table S1.** Top 100 up-regulated genes associated with radioresistance in HNC cells.

| Gene Symbol | Description | P-value | FC (RR vs. PT) |
| --- | --- | --- | --- |
| KRT6A | keratin 6A | 0.03014 | 16.404 |
| NDRG1 | N-myc downstream regulated 1 | 0.00137 | 5.998 |
| LAMA3 | laminin, alpha 3 | 0.02380 | 4.997 |
| GJA1 | gap junction protein alpha 1 | 0.03729 | 4.635 |
| CD55 | CD55 molecule | 0.02070 | 4.371 |
| AHNAK2 | AHNAK nucleoprotein 2 | 0.00056 | 4.220 |
| DDIT4 | DNA damage inducible transcript 4 | 0.02641 | 4.099 |
| FZD10 | frizzled class receptor 10 | 0.02426 | 4.036 |
| ENO2 | enolase 2 | 0.02173 | 4.016 |
| BNIP3L | BCL2 interacting protein 3 like | 0.02336 | 3.861 |
| FST | follistatin | 0.00390 | 3.837 |
| CDH11 | cadherin 11 | 0.04307 | 3.542 |
| P4HA1 | prolyl 4-hydroxylase subunit alpha 1 | 0.03450 | 3.512 |
| GEM | GTP binding protein overexpressed in skeletal muscle | 0.02859 | 3.376 |
| KDM5B | lysine demethylase 5B | 0.00022 | 3.366 |
| PLOD2 | procollagen-lysine, 2-oxoglutarate 5-dioxygenase 2 | 0.00002 | 3.355 |
| ITGA6 | integrin alpha 6 | 0.04988 | 3.279 |
| KDM3A | lysine demethylase 3A | 0.04422 | 3.255 |
| LOX | lysyl oxidase | 0.04873 | 3.235 |
| STC2 | stanniocalcin 2 | 0.00162 | 3.163 |
| IGF2BP3 | insulin-like growth factor 2 mRNA binding protein 3 | 0.01874 | 3.044 |
| CEMIP | cell migration inducing protein, hyaluronan binding | 0.02912 | 3.030 |
| LAMC2 | laminin, gamma 2 | 0.00877 | 3.017 |
| LBH | limb bud and heart development | 0.04099 | 2.981 |
| NT5E | 5'-nucleotidase ecto | 0.00666 | 2.737 |
| IDH2 | isocitrate dehydrogenase 2 (NADP+), mitochondrial | 0.02358 | 2.685 |
| GLIPR1 | GLI pathogenesis related 1 | 0.01261 | 2.653 |
| TCFL5 | transcription factor like 5 | 0.04947 | 2.610 |
| SLC2A1 | solute carrier family 2 member 1 | 0.04175 | 2.560 |
| MYL9 | myosin light chain 9 | 0.03010 | 2.514 |
| P4HA2 | prolyl 4-hydroxylase subunit alpha 2 | 0.01795 | 2.511 |
| DNAJB9 | DnaJ heat shock protein family (Hsp40) member B9 | 0.01172 | 2.493 |
| CTSV | cathepsin V | 0.00344 | 2.456 |
| ARNTL | aryl hydrocarbon receptor nuclear translocator-like | 0.00597 | 2.408 |
| TGFB1I1 | transforming growth factor beta 1 induced transcript 1 | 0.03052 | 2.400 |
| SERPINB2 | serpin family B member 2 | 0.00711 | 2.379 |
| CPD | carboxypeptidase D | 0.02840 | 2.337 |
| TSC22D1 | TSC22 domain family member 1 | 0.00004 | 2.302 |
| FLNB | filamin B | 0.03441 | 2.276 |
| SGK1 | serum/glucocorticoid regulated kinase 1 | 0.00700 | 2.228 |
| ITGB4 | integrin beta 4 | 0.00274 | 2.221 |
| VEGFA | vascular endothelial growth factor A | 0.04618 | 2.189 |
| NEAT1 | nuclear paraspeckle assembly transcript 1 (non-protein coding) | 0.02280 | 2.186 |
| CLK1 | CDC like kinase 1 | 0.00002 | 2.132 |
| DUSP4 | dual specificity phosphatase 4 | 0.04367 | 2.130 |
| CYP1A1 | Cytochrome P450 family 1 subfamily A member 1 | 0.03330 | 2.121 |
| CLU | clusterin | 0.02228 | 2.118 |
| TMEM260 | transmembrane protein 260 | 0.00185 | 2.115 |
| CCDC92 | coiled-coil domain containing 92 | 0.00514 | 2.111 |
| PRSS8 | protease, serine, 8 | 0.01863 | 2.105 |
| CAST | calpastatin | 0.00036 | 2.095 |
| IL6 | interleukin 6 | 0.02283 | 2.066 |
| ITGB1 | integrin beta 1 | 0.00936 | 2.060 |
| MARCH6 | membrane associated ring-CH-type finger 6 | 0.04096 | 2.042 |
| CITED2 | Cbp/P300 interacting transactivator with Glu/Asp rich carboxy-terminal domain 2 | 0.02806 | 2.041 |
| SLC9A6 | solute carrier family 9 member A6 | 0.03015 | 2.039 |
| BHLHE40 | basic helix-loop-helix family member E40 | 0.00570 | 2.038 |
| NFIL3 | nuclear factor, interleukin 3 regulated | 0.03740 | 2.025 |
| JARID2 | Jumonji and AT-rich interaction domain containing 2 | 0.01398 | 2.021 |
| TRIB1 | tribbles pseudokinase 1 | 0.01923 | 2.019 |
| RGS2 | regulator of G-protein signaling 2 | 0.04540 | 1.994 |
| KCNK1 | potassium two pore domain channel subfamily K member 1 | 0.03030 | 1.987 |
| LAMP2 | lysosomal associated membrane protein 2 | 0.00330 | 1.987 |
| PDIA5 | protein disulfide isomerase family A member 5 | 0.04252 | 1.982 |
| DBN1 | drebrin 1 | 0.02794 | 1.974 |
| PLEC | plectin | 0.03824 | 1.971 |
| SERINC3 | serine incorporator 3 | 0.01848 | 1.967 |
| CARS | cysteinyl-tRNA synthetase | 0.02613 | 1.966 |
| SLC27A3 | solute carrier family 27 member 3 | 0.01987 | 1.960 |
| BZW1 | basic leucine zipper and W2 domains 1 | 0.04300 | 1.947 |
| HMOX1 | heme oxygenase 1 | 0.02686 | 1.943 |
| PODXL | podocalyxin like | 0.00164 | 1.931 |
| BTD | biotinidase | 0.02085 | 1.929 |
| CLN5 | ceroid-lipofuscinosis, neuronal 5 | 0.00783 | 1.928 |
| CCDC47 | coiled-coil domain containing 47 | 0.02784 | 1.915 |
| LSR | lipolysis stimulated lipoprotein receptor | 0.04897 | 1.911 |
| SLC1A1 | solute carrier family 1 member 1 | 0.00710 | 1.894 |
| GLG1 | golgi glycoprotein 1 | 0.02973 | 1.878 |
| GPRC5A | G protein-coupled receptor class C group 5 member A | 0.02602 | 1.862 |
| BICD2 | BICD cargo adaptor 2 | 0.00176 | 1.851 |
| IRF6 | interferon regulatory factor 6 | 0.01577 | 1.848 |
| ERP44 | endoplasmic reticulum protein 44 | 0.00377 | 1.839 |
| TTC17 | tetratricopeptide repeat domain 17 | 0.02126 | 1.833 |
| IRS2 | insulin receptor substrate 2 | 0.01757 | 1.818 |
| SLC2A3 | solute carrier family 2, member 3 | 0.01409 | 1.816 |
| MMP12 | matrix metallopeptidase 12 | 0.00149 | 1.816 |
| BLMH | bleomycin hydrolase | 0.02130 | 1.812 |
| FKBP9 | FK506 binding protein 9 | 0.01088 | 1.793 |
| CEBPG | CCAAT/enhancer binding protein gamma | 0.00181 | 1.790 |
| KLF6 | Kruppel like factor 6 | 0.02730 | 1.786 |
| EXT1 | exostosin glycosyltransferase 1 | 0.00057 | 1.782 |
| FAM162A | family with sequence similarity 162 member A | 0.00745 | 1.762 |
| MARCKSL1 | MARCKS like 1 | 0.01869 | 1.754 |
| PTPRR | protein tyrosine phosphatase, receptor type R | 0.03871 | 1.743 |
| MYH9 | myosi heavy chain 9 | 0.00539 | 1.740 |
| CLCN3 | chloride voltage-gated channel 3 | 0.00316 | 1.730 |
| RRAD | Ras-related associated with diabetes | 0.04797 | 1.728 |
| SARAF | store-operated calcium entry associated regulatory factor | 0.01481 | 1.701 |
| FOSL2 | FOS Like 2, AP-1 transcription factor subunit | 0.01911 | 1.693 |
| IGF1R | insulin like growth factor 1 receptor | 0.00260 | 1.689 |

Abbreviations: FC, fold change; RR, radioresistant subline; Pt, parental cell line

**Table S2.** Top 50 down-regulated genes associated with radioresistance in HNC cells.

| Gene Symbol | Description | P-value | FC (RR vs. PT) |
| --- | --- | --- | --- |
| TNFAIP2 | TNF alpha induced protein 2 | 0.01907 | -3.513 |
| FGF4 | fibroblast growth factor 4 | 0.01296 | -2.362 |
| MRPS30 | mitochondrial ribosomal protein S30 | 0.02855 | -2.321 |
| MBNL1 | muscleblind like splicing regulator 1 | 0.01896 | -2.168 |
| ARHGDIA | Rho GDP dissociation inhibitor alpha | 0.04966 | -2.121 |
| HOXD10 | homeobox D10 | 0.01352 | -2.095 |
| SUCLA2 | succinate-CoA ligase ADP-forming beta subunit | 0.02039 | -2.041 |
| MRPL39 | mitochondrial ribosomal protein L39 | 0.00288 | -2.020 |
| DDAH1 | dimethylarginine dimethylaminohydrolase 1 | 0.04301 | -2.018 |
| KRAS | Kirsten rat sarcoma viral oncogene homolog | 0.02666 | -1.966 |
| FAR2 | fatty acyl-CoA reductase 2 | 0.00303 | -1.945 |
| ASF1A | anti-silencing function 1A histone chaperone | 0.00955 | -1.940 |
| CCT8 | chaperonin containing TCP1 subunit 8 | 0.02721 | -1.909 |
| PRKRA | protein kinase, interferon-inducible double stranded RNA dependent activator | 0.01894 | -1.899 |
| MED28 | mediator complex subunit 28 | 0.04468 | -1.880 |
| GCH1 | GTP cyclohydrolase 1 | 0.03124 | -1.858 |
| PAK6 | p21 (RAC1) activated kinase 6 | 0.00002 | -1.854 |
| USP16 | ubiquitin specific peptidase 16 | 0.02968 | -1.852 |
| DCPS | decapping enzyme, scavenger | 0.04222 | -1.846 |
| ATP5J | ATP synthase, H+ transporting, mitochondrial Fo complex subunit F6 | 0.02329 | -1.796 |
| MTX2 | metaxin 2 | 0.00121 | -1.793 |
| BRIX1 | BRX1, biogenesis of ribosomes | 0.02598 | -1.775 |
| NPRL3 | NPR3 like, GATOR1 complex subunit | 0.02506 | -1.769 |
| ZC3H15 | zinc finger CCCH-type containing 15 | 0.01948 | -1.767 |
| ARFRP1 | ADP-ribosylation factor related protein 1 | 0.02212 | -1.757 |
| AGFG1 | ArfGAP with FG repeats 1 | 0.03022 | -1.746 |
| DLAT | dihydrolipoamide S-acetyltransferase | 0.04145 | -1.744 |
| SMC3 | structural maintenance of chromosomes 3 | 0.00011 | -1.743 |
| TRAFD1 | TRAF-type zinc finger domain containing 1 | 0.00040 | -1.738 |
| RFC5 | replication factor C subunit 5 | 0.04027 | -1.732 |
| RANGRF | RAN guanine nucleotide release factor | 0.03404 | -1.727 |
| PPP4R4 | protein phosphatase 4 regulatory subunit 4 | 0.00087 | -1.726 |
| GYG1 | glycogenin 1 | 0.04912 | -1.713 |
| UBE2J1 | ubiquitin conjugating enzyme E2 J1 | 0.02262 | -1.712 |
| DROSHA | Drosha ribonuclease III | 0.02288 | -1.708 |
| TBL1XR1 | transducin beta-like 1 X-linked receptor 1 | 0.03671 | -1.704 |
| ACD | ACD, shelterin complex subunit and telomerase recruitment factor | 0.01639 | -1.698 |
| HMGN1 | high mobility group nucleosome binding domain 1 | 0.01251 | -1.694 |
| STRA13 | stimulated by retinoic acid 13 | 0.00024 | -1.692 |
| ASUN | asunder spermatogenesis regulator | 0.01574 | -1.689 |
| MPHOSPH9 | M-phase phosphoprotein 9 | 0.00678 | -1.674 |
| NFIB | nuclear factor I B | 0.00318 | -1.670 |
| OLFM4 | olfactomedin 4 | 0.00029 | -1.667 |
| LRRC40 | leucine rich repeat containing 40 | 0.02281 | -1.665 |
| IDH3A | isocitrate dehydrogenase 3 (NAD+) alpha | 0.04665 | -1.659 |
| SNX1 | sorting nexin 1 | 0.04918 | -1.653 |
| DCLRE1A | DNA cross-link repair 1A | 0.00054 | -1.648 |
| SEC13 | SEC13 homolog, nuclear pore and COPII coat complex component | 0.00675 | -1.645 |
| HNRNPD | heterogeneous nuclear ribonucleoprotein D | 0.00453 | -1.644 |
| PLAU | plasminogen activator, urokinase | 0.03338 | -1.644 |

Abbreviations: FC, fold change; RR, radioresistant subline; Pt, parental cell line

**Table S3.** Correlative expressions of the 7 molecules in HNC patients receiving radiotherapy.

| Gene | IGF1R | LAMC2 | ITGA6 | ITGB1 | ITGB4 | LAMA3 | IL6 |
| --- | --- | --- | --- | --- | --- | --- | --- |
| IGF1R | - | - | - | - | - | - | - |
| LAMC2 | 0.4501 (<0.0001) | - | - | - | - | - | - |
| ITGA6 | 0.4739 (<0.0001) | 0.7674 (<0.0001) | - | - | - | - | - |
| ITGB1 | 0.3874 (<0.0001) | 0.7000 (<0.0001) | 0.6133 (<0.0001) | - | - | - | - |
| ITGB4 | 0.3332 (0.0030) | 0.6939 (<0.0001) | 0.6682 (<0.0001) | 0.4466 (<0.0001) | - | - | - |
| LAMA3 | 0.4112 (<0.0001) | 0.8696 (<0.0001) | 0.7789 (<0.0001) | 0.6635 (<0.0001) | 0.6663 (<0.0001) | - | - |
| IL6 | 0.1173 (0.0159) | 0.2726 (<0.0001) | 0.1813 (0.0004) | 0.2451 (<0.0001) | 0.0679 (0.1076) | 0.198 (0.0001) | - |

The result was analyzed by Spearman’s correlation test.
